# Investigating the role of P38, JNK and ERK in LPS induced hippocampal insulin resistance and spatial memory impairment: effects of insulin treatment

**DOI:** 10.17179/excli2018-1387

**Published:** 2018-08-20

**Authors:** Parisa Iloun, Zahra Abbasnejad, Mahyar Janahmadi, Abolhassan Ahmadiani, Rasoul Ghasemi

**Affiliations:** 1 Neuroscience Research Center, Shahid Beheshti University of Medical Sciences, Tehran, Iran; 2 Department of Physiology, Faculty of Medicine, Shahid Beheshti University of Medical Sciences, Tehran, Iran; 3 Neurophysiology Research Center, Shahid Beheshti University of Medical Sciences, Tehran, Iran

**Keywords:** insulin, Alzheimer disease, insulin receptor substrate-1, insulin resistance, neuro-inflammation, MAPK

## Abstract

Despite the consensus that neuro-inflammation and insulin resistance (IR) are two hallmarks of Alzheimer disease (AD), the molecular mechanisms responsible for the development of IR remain uncharacterized. MAPKs are signaling molecules that are implicated in the pathology of AD and have a role in IR development. Given that inflammatory mediators are shown to interfere with insulin signaling pathway in different cell types, the present work aimed to investigate whether neuro-inflammation induced memory loss is associated with hippocampal IR and whether insulin treatment protects against this IR. Subsequently, possible roles of MAPKs in this situation were investigated. Male Wistar rats were cannulated, and LPS (15 µg, day 0), insulin (3 mU) or saline (vehicle) were administered intra-cerebroventricularly (ICV) (days 1-6). Spatial memory performance was assessed during days 7-10 by Morris Water Maze test. Consequently, analysis of the amount of hippocampal phosphorylated forms of P38, JNK, ERK, IRS1 (ser307) and Akt (ser473) were done by Western blot. The outcomes indicated that while LPS induced memory loss and hippocampal IR (shown by elevated IRS1 and decreased Akt phosphorylation), insulin treatment nullified these effects. Molecular results also showed that LPS mediated IR and memory loss are associated with P38 but not JNK and ERK activation; this P38 activation was reversed by insulin treatment. These observations implied that one of the ways by which neuro-inflammation participates in AD is via induction of IR. It seems that this IR is mainly mediated by P38. Therefore, P38 could be considered as a molecular target for preventing IR development.

## Introduction

After decades of arduous studies, it is now evident that insulin signaling is a crucial component in the physiology of the central nervous system (Ghasemi et al., 2013[17][18]). Meanwhile, disruption of neuronal insulin signaling or insulin resistance (IR) is also an undeniable pathologic feature of neurodegenerative diseases such as Alzheimer disease (AD) (Ghasemi et al., 2013[17]); however, the exact mechanism responsible for the development of this central IR is still unclear. AD, as the main neurodegenerative disease causing adult dementia, is distinguished by the amyloid beta (Aβ) accumulation in extracellular plaques and intracellular neurofibrillary tangles that promote neuro-inflammatory processes (Zaky et al., 2014[47]). Moreover, proofs dating back to over 100 years have revealed a correlation between inflammation and IR (Williamson, 1901[44]). Since then, evidence has continued to mount showing that in both peripheral and central tissues, inflammation could interfere with insulin signaling pathway and promote IR (Arruda et al., 2011[2]; Hotamisligil et al., 1993[25]; Milanski et al., 2012[33]). So, it is conceivable to assume that inflammatory processes seen in the AD pathology may be associated with the development of IR in the affected structures of the brain, particularly in the hippocampus. In other words, the induction of central IR could be one of the ways by which neuro-inflammation exacerbates the pathology of AD. Considering this probability, the question of how insulin signaling is damaged in the central nervous system might be inclined toward how inflammation promotes IR.

Mitogen activated protein kinases (MAPKs) are the family of kinases, including JNK, P38 and ERK, which are activated by various extracellular stimuli and stresses such as inflammatory cytokines and they participate in the cellular response to these stimuli (Jin et al., 2006[27]; Koistinaho and Koistinaho, 2002[30]) . Several lines of evidence have shown that these kinases are associated with the pathology of AD, and a relationship exists between features of AD and these kinases (Kim and Choi, 2010[28]). For example, MAPKs members are shown to participate in tau hyperphosphorylation and Aβ pathology (Cho et al., 2007[8]; Colombo et al., 2009[9]; Kirouac et al., 2017[29]). Moreover, peripheral evidence has shown that activation of these kinases could interfere with insulin signaling. Therefore, the present work aimed to investigate whether the induction of hippocampal IR is a deleterious effect of LPS mediated neuro-inflammation that eventually produces memory impairment. In addition, it aimed to investigate how neuro-inflammation promotes this IR; if the members of MAPK signaling pathway are involved in this process, and eventually whether central insulin treatment could protect against inflammatory induced hippocampal damages.

## Materials and Methods

### Animals

Adult male Wistar rats weighing 230-280 g obtained from the animal house of the Neuroscience Research Center, Shahid Beheshti University of Medical Sciences were used in this study. The animals were kept in Plexiglas cages in groups of 2-3 per cage under standard laboratory conditions including temperature of 25 ± 2 ºC and controlled light (12 h light-dark cycle, lights on at 07:00 a.m.). Rats were provided with food and water *ad libitum*. The animal care was according to the NIH Guide for the care and use of laboratory animals, and the entire protocols were made to decrease the number of animals used and indeed alleviate their suffering during the experiment. The ethics committee of Shahid Beheshti University of Medical Sciences approved the experimental protocols (Code: IR.SBMU.RAM.REC.1394.158).

### Stereotaxic surgery 

Randomly selected adult male rats (n=8-12 per each group) were stereotaxically implanted with a stainless steel guide cannula (22-gauge) into the right lateral ventricle (AP:-0.84, ML: 1.6 and DV: 3) according to the atlas coordinates of Paxinos. The rats were anesthetized with IP injection of mixed Ketamine (100 mg/kg) and Xylazine (10 mg/kg), and the canulae were anchored to the skull using stainless screws and acrylic cement.

### Drug administration

The ICV microinjection of drugs was fulfilled using a Hamilton syringe (5 µl) connected to the injection cannula through a piece of polyethylene tube. This injection cannula was designed to extend at least 0.5 mm below the tip of the guide cannula and this cannula was left in place for additional 5 min to minimize the backflow of the drug. Microinjection of a single dose (15 µg/ 3µl) of LPS (L2882, Sigma) or its solvent (saline) was performed right after the surgery (day 0), and ICV administration of 3 mU insulin (based on a previous study (Bahramian et al., 2016[3])) or its vehicle (saline) (1 µl/day ICV) were done at exactly six days after surgery (days 1-6). These microinjections were administered at a constant speed of 0.5 µl/min. MWM test was conducted 24 h after the last microinjection (from day 7-10).

### Behavioral test

#### Morris water maze apparatus

In order to assess the spatial memory and learning, MWM apparatus was used. This apparatus is composed of a black circular pool with a diameter of 140 cm and a height of 70 cm, filled with 20 ± 1 ºC water to a depth of 25 cm and a transparent platform (10 cm diameter, 23 cm height) which is placed in a fixed position, 2 cm beneath the water surface. The maze was divided into four equal size quadrants (geographically NW, SW, NE, and SE), and start locations were set in each quadrant. In addition, fixed immovable visual cues were provided in the room (i.e. camera on the ceiling, a door, a column, bookshelves and paper shapes stuck to the walls). A CCD camera was positioned above the pool for recording behavioral sessions and the data were sent to an automated tracking system (Noldus, EthoVision XT 11), then intended parameters (i.e. Latency to reach the hidden or visible platform and the swimming speed) were obtained and analyzed by a software. The whole protocol in MWM was done under four days. In the first three constructive days, the invisible platform was fixed in the center of the SW quadrant and in each day, the rats underwent four trials of learning, beginning from different start points. Before commencing the first trial on the first day, rats were placed on a submerged platform for 20 s. Then in each trial, the rat was given 90 s to locate the hidden platform and allowed to rest on this platform for 20 s until the onset of next trial. The experimenter directed rats that could not locate the platform within 90 s. The latency to find the platform was extracted by the software. On the fourth day, a visible test was conducted. In this test, the hidden platform was replaced by a visible platform, which was covered by a piece of aluminium foil located in the opposite quadrant; rat motivation, visual ability and sensorimotor coordination were then tested in four trials.

### Tissue preparation 

On the last day of the behavioral test and shortly after complementing the visible trials, CO_2_ inhalation was used in the entire animals to anesthetized them; consequently, they were decapitated and the hippocampi were immediately isolated. Then, they were snap frozen in liquid nitrogen and eventually stored at a temperature of -80 ºC until biochemical analysis. 

### Western blot analysis

Western immunoblotting was used to determine expression and phosphorylation of targeted proteins. Accordingly, the hippocampi of the rats (n =4 assigned randomly to each group) were first homogenized with cold RIPA lysis buffer containing protease and phosphatase inhibitor cocktail. Then the lysates were centrifuged at 14,000 g for 30 min at 4 °C, and protein containing supernatants were collected. The protein concentration of samples was assessed by Lowry method. Base on the attained results, equal amounts from each sample were boiled in 2x sample buffer for 5 min and were loaded and electrophoresed in SDS-PAGE. In the next step, separated proteins were transferred to a polyvinylidene difluoride (PVDF) membrane (Millipore). After blocking in 5 % BSA for one hour, the membranes were incubated with primary antibodies (phospho-P38 (9211), phospho-JNK (4671), phospho-ERK (4377), total P38 (8690), total JNK (9252), total ERK (4695), phospho-IRS-1(ser307) (2381), total IRS-1 (2382), phospho-Akt(ser473) (4060) and total Akt (9272) (Cell Signaling Technology) overnight at 4 °C. The next day, after washing with TBS-T, the membranes were incubated for 2 h at room temperature with horseradish peroxidase-conjugated anti-rabbit antibody (7074, Cell Signaling Technology). Immunoreactive bands were visualized using chemiluminescent detection (ECL select, RPN2235, GE healthcare) kit. Eventually, the radiographic films were scanned and integrated densities of bands were measured by Image J software and with the density of each band normalized to control. Thereafter, the normalized data from each phosphorylated band was divided into its total antibodies in the same blot and these normalized ratios (n=4 in each group) were analyzed by one-way analysis of variance (ANOVA). 

### Data analysis

Data obtained from three days of learning experiments were analyzed by repeated measures ANOVA. Results obtained in the visible test and swimming speed, as well as molecular data were analyzed by one-way analysis of variance (ANOVA). Tukey's post-hoc test was used for intragroup comparisons. All results were presented as means ± SEM. In all statistical comparisons, P < 0.05 was considered as the significant difference. All raw data of the present manuscript are given in Supplementary Tables 1-9 

 .

## Results

### Insulin nullifies LPS induced spatial memory impairment

**Figure 1:**
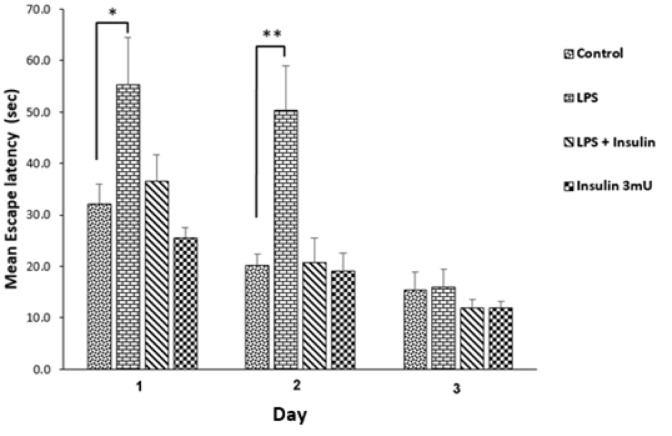
The effect of vehicle, LPS or (and) insulin administration on water maze spatial learning and memory. Repeated measure ANOVA showed an overall significant difference in this graph (P = 0. 002). The escape latency to the hidden platform during days 1-3 of training are compared day by day. Data are represented as mean ± SEM. * P < 0.05 and ** P < 0.01 represent the significant difference between control and other groups in each day.

**Figure 2:**
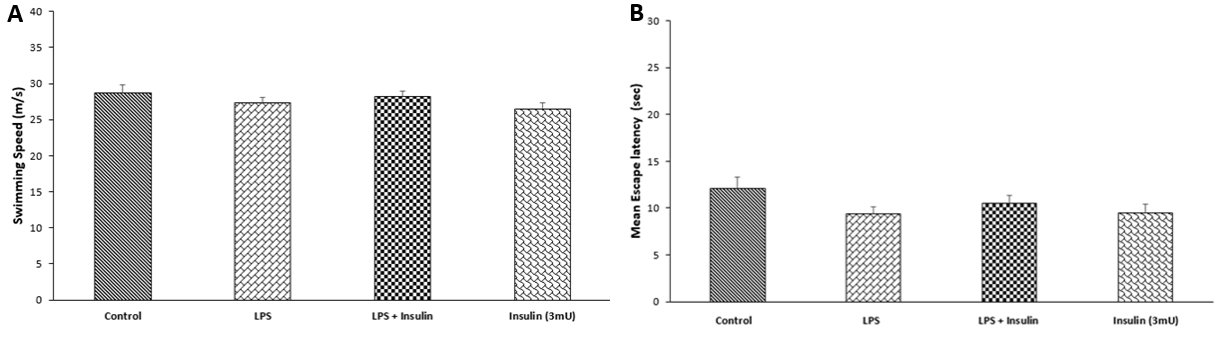
The effect of ICV saline, LPS or (and) insulin administration on animals' swimming speed (A) and escape latency to reach the visible platform (B). Data are represented as mean ± S.E.M. Swimming speed and escape latency to the visible platform did not show significant difference between groups.

In the first step, investigation on whether central (ICV) injection of LPS could bring about spatial learning and memory loss was carried out; if so, can insulin protect against it or not. The overall results of spatial learning and memory obtained in MWM test are presented in Figures 1-2(Fig. 1)(Fig. 2). The effects of LPS or (and) insulin (3 mU) administration on escape latency to reach the hidden platform is presented in Figure 1(Fig. 1). As evident in the learning patterns of animals in Figure 1(Fig. 1), a negative linear correlation existed between escape latency and training days in all groups (reduction of escape latencies on day 2/day 1 and day 3/day 1). This indicated that all groups learnt the platform location; however, LPS administration significantly slowed down the learning capability, particularly from day 1 to day 2. Repeated measures ANOVA analysis of escape latency in 3 training days showed a significant difference between groups (P = 0. 002, F _(3, 37)_ = 5.910). The Post-hoc Tukey's test following repeated measure analysis was used for intragroup comparison and outcomes indicated that escape latency in LPS receiving group was significantly more than that of vehicle receiving group (P = 0.009). This post-hoc test also revealed that treatment with 3 mU insulin for six days reversed this neuro-inflammation induced behavioral impairment (P = 0.014, significant difference between LPS group and LPS + Insulin group).

To compare how animals behaved in distinct days of training, mean escape latency of groups on each day were analyzed by one-way ANOVA. From the results, a significant difference existed between groups on the first and second days (Day 1: P = 0. 008, F _(3,37) _= 4.595, Day 2: P < 0. 001, F _(3,37) _= 8.059), but one-way ANOVA test showed that no significant difference existed between groups on the third day (P = 0. 652, F _(3,37)_ = 0.549). The post hoc Tukey test was used following one-way ANOVA, and results showed that in comparison with the control group, LPS significantly elevated escape latency in the first two training days; on the other hand, insulin treatment prevented this impairing effect of LPS (the level of significance is shown by asterisks in Figure 1(Fig. 1)).

### Behavioral effects of treatments were not achieved via motivational effect or by affecting sensorimotor function of animals

To investigate whether the treatments affected sensorimotor function and/or animal's motivation, we compared the mean swimming speed during MWM and on the last day of test, a visible platform test was conducted. Figure 2A(Fig. 2) depicts the mean swimming speed of the animals during MWM test. One-way ANOVA analysis showed no significant differences between groups (P = 0.4445, F_(3,37) _= 0.9120). Figure 2B(Fig. 2) shows the effect of treatments on escape latency to the visible platform on last day of the test. One-way ANOVA of escape latency to the visible platform also did not present significant differences between groups (P = 0.7266, F_ (3, 37)_ = 0.4387). 

### Central LPS administration hampered insulin signaling transduction and insulin treatment reverses these effects

**Figure 3:**
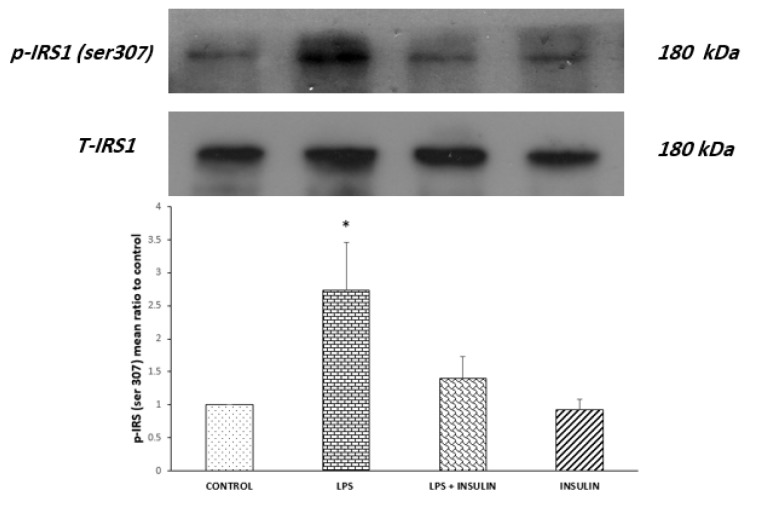
Western blot analysis showing the effects of ICV microinjection of the vehicle, LPS and (or) insulin on the mean ratio of normalized phospho-IRS-1 (Ser307) to total IRS-1 in the hippocampi of rats. Representative immunoblots are shown in the upper panel. *P < 0.05 represents the difference between control and the LPS receiving group (n = 4 for each group).

Molecular experiments were conducted on the hippocampi of rats to investigate the effect of LPS mediated neuro-inflammation and/or central insulin treatment on the activity of MAPK and insulin signaling pathways. In order to examine if LPS induced behavioral deficits were associated with alteration in hippocampal insulin signaling transduction, the amounts of inhibitory phosphorylation of IRS-1 in serine 307 residue and activating phosphorylation of Akt at serine 473 were evaluated by Western blot experiments. Figure 3(Fig. 3) shows the results of Western blot analysis of inhibitory phosphorylation of IRS-1 in serine 307. In Western blotting, antibodies against phospho IRS-1 (Ser307) and total IRS-1 detected a band at 180 kDa. 

In order to analyze the mean normalized ratio of phospho-IRS-1 (Ser307) to total form of IRS-1, one-way ANOVA was used, and the results showed a significant difference between groups (P = 0.0286, F_(3,12)_ = 4.277). In the post-hoc analysis with Tukey's test, a comparison of the normalized extent of IRS-1 phosphorylation in the drug with the control was made, and results revealed that LPS microinjection significantly increased IRS-1 phosphorylation at serine 307 position (P < 0.05); and six days of treatment with 3 mU insulin halted this LPS induced IRS-1 phosphorylation. 

**Figure 4:**
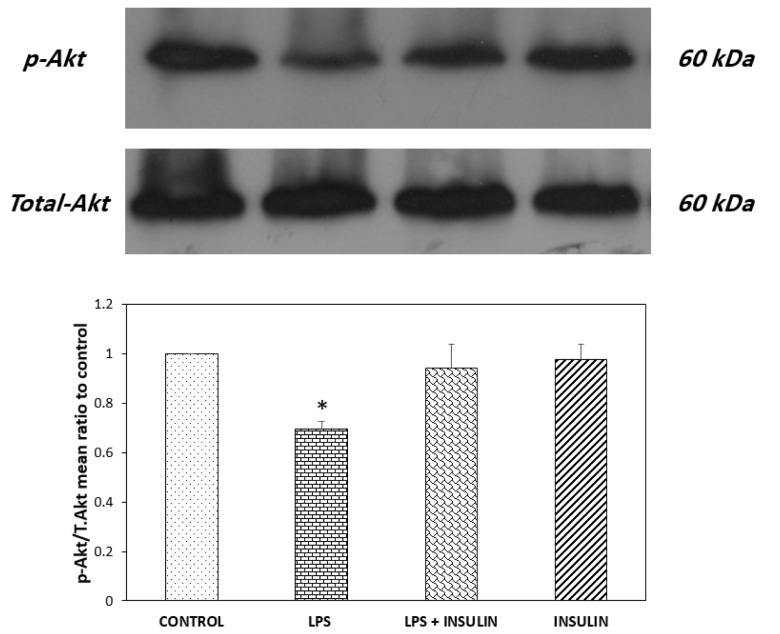
Western blot analysis showing the effects of ICV microinjection of the vehicle, LPS and (or) insulin on the ratio of phosphorylated Akt (Ser473) to total Akt protein in the hippocampi of rats. Representative immunoblots of phospho and total Akt are depicted in the upper panel. *P < 0.05 represents the difference between control and the LPS receiving group (n = 4 for each group).

IRS-1 has several negative and positive phosphorylation sites. Therefore, just assessing IRS-1 phosphorylation at each site does not definitely mean that insulin signaling is inhibited or activated. Then, in order to see if this elevated phosphorylation of IRS-1 in its negative site (S307) is really accompanied with reduced insulin signaling or just makes it harder, the phosphorylation of Akt on its activation site was analyzed by Western blot. The outcomes indicating the normalized ratio of phosphorylated (Ser473) to the total form of Akt in the hippocampus are depicted in Figure 4(Fig. 4). In Western blotting, antibodies against phospho and total Akt detected a band at 60 kDa. One-way ANOVA showed a significant difference between groups (P = 0.0011, F _(3,12) _= 10.485). To examine the differences between the groups, post-hoc analysis with Tukey's test was conducted and the results showed that in comparison to control (vehicle receiving) group, LPS microinjection significantly suppressed hippocampal Akt activity (P < 0.05); however, six days of insulin treatment nullified this LPS-induced decrement of Akt activation.

### While central LPS administration increased P38 activity and insulin treatment negated this effect, these treatments had no detectable effects on JNK and ERK

**Figure 5:**
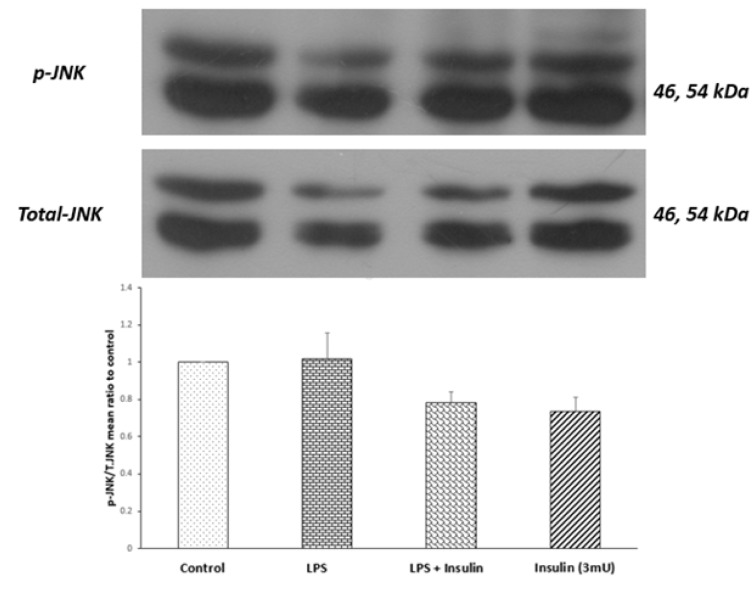
Western blot analysis showing the effects of ICV microinjection of the vehicle, LPS and (or) insulin on the ratio of phosphorylated JNK to total JNK protein in the hippocampi of rats. Representative immunoblots are depicted in the upper panel. One-way ANOVA did not show any significant differences (n = 4 for each group).

To explore if LPS mediated behavioral deficits and decreased hippocampal insulin signaling had any correlation with the activity of MAPK members, the amounts of ERK phosphorylation, JNK, and hippocampal P38 were evaluated by Western blot study. The outcomes indicating the normalized ratio of phosphorylated to total form of JNK in the hippocampi are presented in Figure 5(Fig. 5). Antibodies against phospho and total JNK detected two bands with an apparent molecular weight of 46 and 54 kDa. There was no significant difference between groups (P = 0.0705, F _(3,12) _= 3.040) using one-way ANOVA, indicating that central LPS and/or insulin treatment had no detectable effects on JNK activity at the point of our evaluation.

**Figure 6:**
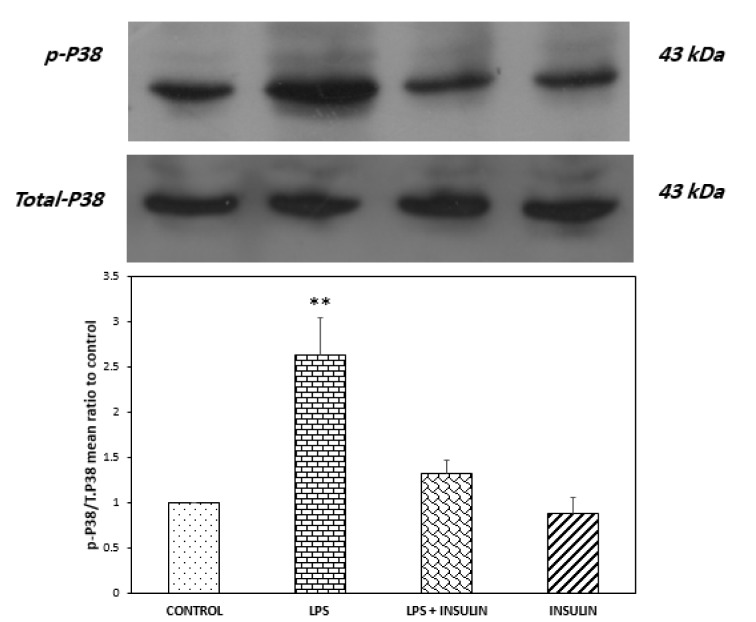
Western blot analysis showing the effects of ICV microinjection of vehicle, LPS and (or) insulin on the ratio of phosphorylated P38 to total P38 protein in the hippocampi of rats. Representative immunoblots for phospho and total P38 are shown in the upper panel. **P < 0.01 represents the difference between control and the LPS receiving group (n = 4 for each group).

Figure 6(Fig. 6) illustrates the analysis of Western blot on the activity of P38 as the ratio of phosphorylated to the total form of P38. Antibodies against phospho and total P38 detected a band at 43 kDa. One-way ANOVA revealed that a significant difference existed between groups (P = 0.0006, F _(3,12)_ = 12.163). In the next step, post-hoc analysis with Tukey's test was performed to compare groups; the results revealed that LPS microinjection caused significant increase in hippocampal P38 activity when compared to the control group (P < 0.01). This analysis also showed that LPS+ insulin group had no significant difference with control group, indicating that six days of insulin treatment prevented LPS-mediated elevation of P38 activity.

**Figure 7:**
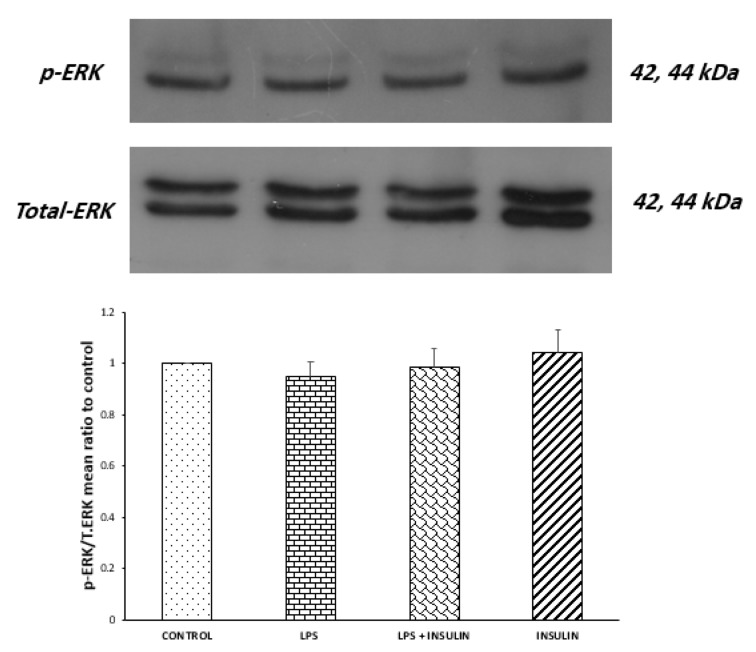
Western blot analysis showing the effects of ICV microinjection of the vehicle, LPS and (or) insulin on the ratio of phosphorylated ERK to total ERK protein in the hippocampi of rats. Representative immunoblots for phospho and total ERK are shown in the upper panel. One-way ANOVA did not show any significant differences (n = 4 for each group).

Figure 7(Fig. 7) shows the results of Western blot analysis on the activity of ERK. Antibodies against phospho and total ERK identified two bands at 42 and 44 kDa. No significant difference was seen between groups (P = 0.7909, F _(3,12) _= 0.3485), indicating that central LPS and/or insulin treatment had no detectable effects on ERK activity at the point of our evaluation. 

## Discussion

This scientific probe aimed to elucidate the relationship between neuro-inflammation, memory loss, insulin signaling and the molecular mechanisms, which contribute to these processes. Neuro-inflammation is a hallmark of AD (Fischer and Maier, 2015[15]) and LPS-induced neuro-inflammation is a common *in vivo* and *in vitro* model in AD studies (reviewed in Zakaria et al. (2017[46])). In order to avoid possible development of tolerance (Shaw et al., 2001[39]) and according to our pilot studies and previous studies (Kolahdooz et al., 2015[31]; Omidbakhsh et al., 2014[34]), for the purpose of this, we chose a single central LPS microinjection (15 µg in 3 µl) to develop a neuro-inflammation induced memory loss. The behavioral results showed that while single LPS administration impaired spatial learning and memory, six days of central insulin treatment (3 mU/day) prevented this learning and memory deterioration. 

Mounting evidence has shown that insulin signaling plays an indispensable role in the physiological functions of the brain and confounding factors which hamper these functions and help to develop central IR, contribute to the physiopathology of neurodegenerative disorders such as AD (reviewed in Ghasemi et al., 2013[17]). Therefore, strategies aimed to restore or compensate insulin signaling are areas of intensive research in AD. Accordingly, Adzovic et al. recently reported that four weeks of central LPS administration significantly impaired memory performance in MWM and the same period of co-treatment with central insulin nullified this memory loss (Adzovic et al., 2015[1]). 

There are differences between this study and ours; first, the duration of LPS injection (four weeks vs. single dose) and second, the duration of insulin treatment (four weeks vs. six days). In addition, in present work, focus was on the molecular mechanisms involved in the interplay between insulin and inflammation and the roles of MAPKs in this process. As mentioned before, when the induction of inflammation by LPS is repeated, some distinct cellular mechanisms are activated which may produce different and even some opposing effects than that of single injection (Shaw et al., 2001[39], 2005[38]). As anticipated and in agreement with the study of Adzovic et al. (2015[1]), as well as our previous work (Ghasemi et al., 2014[20]), results of the present study showed that even shorter periods of insulin treatment (six days vs. four weeks) could also protect against neuro-inflammation mediated behavioral deficits. 

The results of molecular studies showed that a single LPS administration not only deteriorated the spatial memory, but also undermined the insulin signaling strength in the hippocampus. As evident in Figures 3(Fig. 3) and 4(Fig. 4), one dose of central LPS injection significantly increased IRS-1 phosphorylation in ser307 residue and decreased Akt activity (as shown in reduced phosphorylation of Akt in ser473 position). IRS-1 is an adaptor molecule with several tyrosine and serine phosphorylation sites that acts as a substrate for insulin receptor. When phosphorylated on tyrosine residues, IRS-1 recruits signal transducers such as PI3-kinase and then activate PKB/Akt as one of the central components of insulin signaling pathway (reviewed in Gual et al., 2005[22]). The IRS-1 molecule also has several serine sites and their phosphorylation by downstream molecules of insulin signaling pathway provides a physiological feedback mechanism for maintaining insulin signaling fidelity (Boura-Halfon and Zick, 2009[6]). Different cellular stressors could distort this intricate balance between tyrosine and serine phosphorylation and bring about difficulties in insulin signaling or IR (Tanti and Jager, 2009[43]). Accordingly, mounting evidence (mainly peripheral and less central) has revealed that elevated serine phosphorylation by different predisposing factors such as inflammatory mediators and its subsequent reduction of Akt phosphorylation and activity are hallmarks of developing IR (reviewed in Gual et al., 2005[22]; Hemmati et al., 2014[23]). In good agreement, the present results implied that LPS mediated memory loss is associated with the development of hippocampal IR. This assumption is in conformity with peripheral (Hotamisligil et al., 1993[25]; Wu and Ballantyne, 2017[45]) as well as central (Adzovic et al., 2015[1]; Rorato et al., 2017[36]) proofs, indicating that inflammatory processes play an indispensable part in the development of IR. Previous studies have proposed several mechanisms by which neuronal inflammation contributes to neurodegenerative processes of AD. For example, by activating microglia, LPS triggers the release of pro-inflammatory cytokines, chemokines and expression of inducible nitric oxide synthase (iNOS) (Bossu et al., 2012[5]; Rubio-Perez and Morillas-Ruiz, 2012[37]). The release of these inflammatory mediators ultimately triggers oxidative and nitrosative stress that ultimately form a vicious cycle that further exacerbates the condition and finally leads to mitochondrial dysfunction and apoptosis (reviewed in Fischer and Maier, 2015[15]). LPS mediated inflammation also increases glutamate release and triggers excitotoxicity (Espey et al., 1998[13]; Fine et al., 1996[14]), alters BBB integrity and by affecting Aβ transport, promotes formation of Aβ plaque (Erickson et al., 2012[12]; Jaeger et al., 2009[26]). Our results strengthen the assumption that induction of IR is another way by which inflammation promotes AD pathology. Our molecular results also revealed that six days of exogenous insulin post-treatment is potent enough to nullify this hippocampal IR induced by inflammatory processes. Analogous to the current results, it has been demonstrated that chronic insulin infusion could protect insulin signaling pathway against deleterious effects of chronic inflammation (Adzovic et al., 2015[1]). In addition, in an *in vitro* experiment, conducted in primary hippocampal cell culture, it was also observed that insulin reversed Aβ mediated Akt inactivation (Ghasemi et al., 2015[19]). These results emphasize that central insulin treatment strategies, such as intranasal insulin therapy, could be considered as a potential treatment in patients at high risk of developing central IR. 

Finally, one of the main goals in conducting this experiment was to explore the molecular mechanisms distinguishing the development of IR in the brain. Accordingly, we chose to evaluate the activity of JNK, P38 and ERK. These members of MAPK signaling pathway, on one hand, play an extremely essential part in the cellular responses to different stressors such as inflammatory mediators, irradiation, osmotic changes, withdrawal of trophic factors, drugs, temperature and changes in cell shape (Bulavin and Fornace, 2004[7]; Stalheim and Johnson, 2008[41]; Koistinaho and Koistinaho, 2002[30]). On the other hand, increasing proofs obtained mainly from peripheral tissues have shown that these omnipresent kinases participate in the induction of IR (Hemmati et al., 2014[23]). Therefore, it was hypothesized that these kinases might be involved in the interaction between neuro-inflammation, hippocampal IR and memory loss. From the molecular results, concurrent with developing IR and spatial memory deficits, single LPS infusion elevated the activity of hippocampal P38; however, no measurable alteration in the activity of JNK and ERK was seen at the time point of evaluation. It was also observed that insulin treatment dampened this P38 over-activity but had no effect on JNK and ERK activities. These results implied that P38 could be the main molecular link between neuro-inflammatory processes and IR. In a previous study, we reported that repeated intrahippocampal infusion of Aβ impaired spatial memory and elevated the activity of P38, JNK and ERK. Also it was observed that over activation of P38 and ERK, but not JNK, as well as memory loss were prevented by six days of intrahippocampal insulin treatment (Ghasemi et al., 2014[20][21]). Despite some differences, such as elevated JNK and ERK activities seen in Aβ microinjection that may originate from type of toxin and/or time interval between drug infusion and molecular studies, these studies also highlight the idea that P38 is an important contributor in memory deterioration and IR. In support of this suggestion, an *in vitro* study on skeletal muscle cells has shown that TNF-α mediated IR (shown by elevated ser307 phosphorylation of IRS-1) could be blocked by pre-treatment with specific inhibitors of P38 but not inhibitors of JNK and ERK (de Alvaro et al., 2004[10]). Identical outcomes were as well attained in adipocytes (Lorenzo et al., 2008[32]). Some lines of evidence obtained from neuronal tissues also exist, indicating that P38 is a key molecular mediator in neuro-inflammatory processes. Accordingly, P38 is shown to be involved in age-related development of hypothalamic IR (Garcia-San Frutos et al., 2012[16]).

Despite these reports, which support our conclusion regarding the role of P38 in IR, studies are also available that are somehow different, for example, some studies reported that other members of MAPK family (JNK and/or ERK) also take part in the induction of IR. Accordingly, Benzler et al. reported that ICV infusion of JNK inhibitor (SP600125) could ameliorate high-fat diet elicited IR of the hypothalamus (Benzler et al., 2013[4]). Similar results are also available in peripheral tissues such as hepatocytes and pancreatic beta-cells (Solinas et al., 2006[40]). Moreover, an *in vitro* study in adipocytes also showed that inhibition of ERK could prevent IR development (Engelman et al., 2000[11]). This discrepancy could be that members of MAPK family are preferentially activated by distinct stimuli and act differentially at different time courses. As mentioned earlier, in the presence of inflammatory stress, P38 would be the main MAPK that is activated and contributes to the harmful effects of this stress. In line with this view, a study on microglial cells revealed that deletion of TLR2 and TLR4, as essential receptors in the induction of inflammation, prevented Aβ mediated P38 activation. On the other hand, specific inhibitor of P38 (SB203580) was shown to prevent the formation of Aβ mediated reactive oxygen species and phagocytosis (Reed-Geaghan et al., 2009[35]) and LPS-stimulated synthesis of pro-inflammatory cytokines (Herman et al., 2014[24]). These results clearly showed a reciprocal relationship between inflammation and P38 activation. It is noteworthy that time is also a determinant factor for the activity of MAPK members, for example; it is believed that the transient or persistent activation of ERK could produce even two opposing effects (survival vs. apoptosis) (Ghasemi et al., 2014[21]; Subramaniam and Unsicker, 2010[42]). In this regard, in an interesting observation, Engelman et al. demonstrated that in 3T3-L1 adipocytes, TNF-α increases serine phosphorylation of IRS-1 and ERK activity but at different time windows. They observed that while TNF-α transiently activated ERK (less than half an hour), more time was needed for the disturbing effects of TNF-α on insulin signaling to develop (2.5-4 h) (Engelman et al., 2000[11]). Therefore, two main reasons could be proposed to describe why significant changes in the activity of ERK and JNK were not seen. The first probable reason is the type of toxin; LPS, which preferentially and more strongly activates P38 activity. The second one is that if there was any activity in ERK and/or JNK, this activity returned to its basal level so that it was not possible to detect this at the time the molecular studies were conducted (ten days after single LPS injection). Since the present evaluations were carried out in one time point, the current work can neither prove nor disprove the possibility of transient JNK and/or ERK activation. However, it must be emphasized that even if these two MAPK were actually activated by LPS but did not last, the coexistence of P38 and elevated IRS-1 phosphorylation at ser307 implies that in the present paradigm, P38 may act a more significant role in the induction of IR. Because of limitations of *in vivo* studies that all pathways and effectors are not controlled and in order to elucidate the exact contribution of these kinases in induction of IR at different time windows and different stressors, future isolated *in vitro *studies could be helpful.

In conclusion, this study broadens our knowledge about the adverse effects of neuro-inflammatory processes in development and progression of AD. In addition, it emphasized the importance of central IR to this process. These results also provide proofs showing that in inflammatory situations, P38 might be the main MAPK associated with the induction of IR. These data proposed that treatment strategies targeting P38 (such as central insulin replacement) could be considered as a potential therapy against the progression of AD.

## Acknowledgements

The deputy of Shahid Beheshti University of Medical Sciences financially supported the present work. The authors are grateful to the Neuroscience Research Center and Neurophysiology Research Center, Shahid Beheshti University of Medical Sciences for supporting this work. 

## Conflict of interest

The authors declare that they have no conflict of interests.

## Supplementary Material

Supplementary table 1

Supplementary table 2

Supplementary table 3

Supplementary table 4

Supplementary table 5

Supplementary table 6

Supplementary table 7

Supplementary table 8

Supplementary table 9

## References

[1] Adzovic L, Lynn AE, D'Angelo HM, Crockett AM, Kaercher RM, Royer SE (2015). Insulin improves memory and reduces chronic neuroinflammation in the hippocampus of young but not aged brains. J Neuroinflamm.

[2] Arruda AP, Milanski M, Coope A, Torsoni AS, Ropelle E, Carvalho DP (2011). Low-grade hypothalamic inflammation leads to defective thermogenesis, insulin resistance, and impaired insulin secretion. Endocrinology.

[3] Bahramian A, Rastegar K, Namavar MR, Moosavi M (2016). Insulin potentiates the therapeutic effect of memantine against central STZ-induced spatial learning and memory deficit. Behav Brain Res.

[4] Benzler J, Ganjam GK, Legler K, Stohr S, Kruger M, Steger J (2013). Acute inhibition of central c-Jun N-terminal kinase restores hypothalamic insulin signalling and alleviates glucose intolerance in diabetic mice. J Neuroendocrinol.

[5] Bossu P, Cutuli D, Palladino I, Caporali P, Angelucci F, Laricchiuta D (2012). A single intraperitoneal injection of endotoxin in rats induces long-lasting modifications in behavior and brain protein levels of TNF-alpha and IL-18. J Neuroinflamm.

[6] Boura-Halfon S, Zick Y (2009). Phosphorylation of IRS proteins, insulin action, and insulin resistance. Am J Physiol Endocrinol Metab.

[7] Bulavin DV, Fornace AJ (2004). p38 MAP kinase's emerging role as a tumor suppressor. Adv Cancer Res.

[8] Cho HJ, Kim SK, Jin SM, Hwang EM, Kim YS, Huh K (2007). IFN-gamma-induced BACE1 expression is mediated by activation of JAK2 and ERK1/2 signaling pathways and direct binding of STAT1 to BACE1 promoter in astrocytes. Glia.

[9] Colombo A, Bastone A, Ploia C, Sclip A, Salmona M, Forloni G (2009). JNK regulates APP cleavage and degradation in a model of Alzheimer's disease. Neurobiol Dis.

[10] De Alvaro C, Teruel T, Hernandez R, Lorenzo M (2004). Tumor necrosis factor alpha produces insulin resistance in skeletal muscle by activation of inhibitor kappaB kinase in a p38 MAPK-dependent manner. J Biol Chem.

[11] Engelman JA, Berg AH, Lewis RY, Lisanti MP, Scherer PE (2000). Tumor necrosis factor alpha-mediated insulin resistance, but not dedifferentiation, is abrogated by MEK1/2 inhibitors in 3T3-L1 adipocytes. Mol Endocrinol.

[12] Erickson MA, Hartvigson PE, Morofuji Y, Owen JB, Butterfield DA, Banks WA (2012). Lipopolysaccharide impairs amyloid beta efflux from brain: altered vascular sequestration, cerebrospinal fluid reabsorption, peripheral clearance and transporter function at the blood-brain barrier. J Neuroinflamm.

[13] Espey MG, Kustova Y, Sei Y, Basile AS (1998). Extracellular glutamate levels are chronically elevated in the brains of LP-BM5-infected mice: a mechanism of retrovirus-induced encephalopathy. J Neurochem.

[14] Fine SM, Angel RA, Perry SW, Epstein LG, Rothstein JD, Dewhurst S (1996). Tumor necrosis factor alpha inhibits glutamate uptake by primary human astrocytes. Implications for pathogenesis of HIV-1 dementia. J Biol Chem.

[15] Fischer R, Maier O (2015). Interrelation of oxidative stress and inflammation in neurodegenerative disease: role of TNF. Oxid Med Cell Longev.

[16] Garcia-San Frutos M, Fernandez-Agullo T, Carrascosa JM, Horrillo D, Barrus MT, Oliveros E (2012). Involvement of protein tyrosine phosphatases and inflammation in hypothalamic insulin resistance associated with ageing: effect of caloric restriction. Mech Ageing Dev.

[17] Ghasemi R, Dargahi L, Haeri A, Moosavi M, Mohamed Z, Ahmadiani A (2013). Brain insulin dysregulation: implication for neurological and neuropsychiatric disorders. Mol Neurobiol.

[18] Ghasemi R, Haeri A, Dargahi L, Mohamed Z, Ahmadiani A (2013). Insulin in the brain: sources, localization and functions. Mol Neurobiol.

[19] Ghasemi R, Moosavi M, Zarifkar A, Rastegar K, Maghsoudi N (2015). The interplay of Akt and ERK in Aß toxicity and insulin-mediated protection in primary hippocampal cell culture. J Mol Neurosci.

[20] Ghasemi R, Zarifkar A, Rastegar K, Maghsoudi N, Moosavi M (2014). Insulin protects against Aß-induced spatial memory impairment, hippocampal apoptosis and MAPKs signaling disruption. Neuropharmacology.

[21] Ghasemi R, Zarifkar A, Rastegar K, Maghsoudi N, Moosavi M (2014). Repeated intra-hippocampal injection of beta-amyloid 25-35 induces a reproducible impairment of learning and memory: considering caspase-3 and MAPKs activity. Eur J Pharmacol.

[22] Gual P, Le Marchand-Brustel Y, Tanti JF (2005). Positive and negative regulation of insulin signaling through IRS-1 phosphorylation. Biochimie.

[23] Hemmati F, Ghasemi R, Mohamed Ibrahim N, Dargahi L, Mohamed Z, Raymond AA (2014). Crosstalk between insulin and toll-like receptor signaling pathways in the central nervous system. Mol Neurobiol.

[24] Herman AP, Krawczynska A, Bochenek J, Antushevich H, Herman A, Tomaszewska-Zaremba D (2014). Peripheral injection of SB203580 inhibits the inflammatory-dependent synthesis of proinflammatory cytokines in the hypothalamus. Biomed Res Int.

[25] Hotamisligil GS, Shargill NS, Spiegelman BM (1993). Adipose expression of tumor necrosis factor-alpha: direct role in obesity-linked insulin resistance. Science.

[26] Jaeger LB, Dohgu S, Sultana R, Lynch JL, Owen JB, Erickson MA (2009). Lipopolysaccharide alters the blood-brain barrier transport of amyloid beta protein: a mechanism for inflammation in the progression of Alzheimer's disease. Brain Behav Immun.

[27] Jin Y, Fan Y, Yan EZ, Liu Z, Zong ZH, Qi ZM (2006). Effects of sodium ferulate on amyloid-beta-induced MKK3/ MKK6-p38 MAPK-Hsp27 signal pathway and apoptosis in rat hippocampus. Acta Pharmacol Sin.

[28] Kim EK, Choi EJ (2010). Pathological roles of MAPK signaling pathways in human diseases. Biochim Biophys Acta.

[29] Kirouac L, Rajic AJ, Cribbs DH, Padmanabhan J (2017). Activation of Ras-ERK signaling and GSK-3 by amyloid precursor protein and amyloid beta facilitates neurodegeneration in Alzheimer's disease. eNeuro.

[30] Koistinaho M, Koistinaho J (2002). Role of p38 and p44/42 mitogen-activated protein kinases in microglia. Glia.

[31] Kolahdooz Z, Nasoohi S, Asle-Rousta M, Ahmadiani A, Dargahi L (2015). Sphingosin-1-phosphate receptor 1: a potential target to inhibit neuroinflammation and restore the Sphingosin-1-phosphate metabolism. Can J Neurol Sci.

[32] Lorenzo M, Fernandez-Veledo S, Vila-Bedmar R, Garcia-Guerra L, De Alvaro C, Nieto-Vazquez I (2008). Insulin resistance induced by tumor necrosis factor-alpha in myocytes and brown adipocytes. J Anim Sci.

[33] Milanski M, Arruda AP, Coope A, Ignacio-Souza LM, Nunez CE, Roman EA (2012). Inhibition of hypothalamic inflammation reverses diet-induced insulin resistance in the liver. Diabetes.

[34] Omidbakhsh R, Rajabli B, Nasoohi S, Khallaghi B, Mohamed Z, Naidu M (2014). Fingolimod affects gene expression profile associated with LPS-induced memory impairment. Exp Brain Res.

[35] Reed-Geaghan EG, Savage JC, Hise AG, Landreth GE (2009). CD14 and toll-like receptors 2 and 4 are required for fibrillar A{beta}-stimulated microglial activation. J Neurosci.

[36] Rorato R, Borges BC, Uchoa ET, Antunes-Rodrigues J, Elias CF, Elias LLK (2017). LPS-induced low-grade inflammation increases hypothalamic JNK expression and causes central insulin resistance irrespective of body weight changes. Int J Mol Sci.

[37] Rubio-Perez JM, Morillas-Ruiz JM (2012). A review: inflammatory process in Alzheimer's disease, role of cytokines. Sci World J.

[38] Shaw KN, Commins S, O'Mara SM (2005). Cyclooxygenase inhibition attenuates endotoxin-induced spatial learning deficits, but not an endotoxin-induced blockade of long-term potentiation. Brain Res.

[39] Shaw KN, Commins S, O'Mara SM (2001). Lipopolysaccharide causes deficits in spatial learning in the water maze but not in BDNF expression in the rat dentate gyrus. Behav Brain Res.

[40] Solinas G, Naugler W, Galimi F, Lee MS, Karin M (2006). Saturated fatty acids inhibit induction of insulin gene transcription by JNK-mediated phosphorylation of insulin-receptor substrates. Proc Natl Acad Sci.

[41] Stalheim L, Johnson GL, Posas F, Nebreda AR (2008). MAPK kinase kinase regulation of SAPK/JNK pathways. Stress-activated protein kinases.

[42] Subramaniam S, Unsicker K (2010). ERK and cell death: ERK1/2 in neuronal death. FEBS J.

[43] Tanti JF, Jager J (2009). Cellular mechanisms of insulin resistance: role of stress-regulated serine kinases and insulin receptor substrates (IRS) serine phosphorylation. Curr Opin Pharmacol.

[44] Williamson RT (1901). On the treatment of glycosuria and diabetes mellitus with sodium salicylate. Br Med J.

[45] Wu H, Ballantyne CM (2017). Skeletal muscle inflammation and insulin resistance in obesity. J Clin Invest.

[46] Zakaria R, Wan Yaacob WM, Othman Z, Long I, Ahmad AH, Al-Rahbi B (2017). Lipopolysaccharide-induced memory impairment in rats: a model of Alzheimer's disease. Physiol Res.

[47] Zaky A, Mahmoud M, Awad D, El Sabaa BM, Kandeel KM, Bassiouny AR (2014). Valproic acid potentiates curcumin-mediated neuroprotection in lipopolysaccharide induced rats. Front Cell Neurosci.

